# Fabrication of ZnO@MoS_2_ Nanocomposite Heterojunction Arrays and Their Photoelectric Properties

**DOI:** 10.3390/mi11020189

**Published:** 2020-02-12

**Authors:** Hui Wu, Huge Jile, Zeqiang Chen, Danyang Xu, Zao Yi, Xifang Chen, Jian Chen, Weitang Yao, Pinghui Wu, Yougen Yi

**Affiliations:** 1 Joint Laboratory for Extreme Conditions Matter Properties, Southwest University of Science and Technology, Mianyang 621010, China; towuhui@outlook.com (H.W.); wtyao@ustc.edu.cn (W.Y.); 2 School of Science, Huzhou University, Huzhou 313000, China; hgjl@zjhu.edu.cn; 3 Research Center for Photonic Technology, Fujian Key Laboratory for Advanced Micro-nano Photonics Technology and Devices & Key Laboratory of Information Functional Material for Fujian Higher Education, Quanzhou Normal University, Quanzhou 362000, China; czqchem@qztc.edu.cn; 4 College of Science, Zhejiang University of Technology, Hangzhou 310023, China; xudanyang@zjut.edu.cn; 5 College of Physics and Electronics, Central South University, Changsha 410083, China; yougenyi@csu.edu.cn

**Keywords:** ZnO@MoS_2_ heterojunction, hydrothermal, photoanode, photoelectric properties

## Abstract

In this paper, ZnO@MoS_2_ core-shell heterojunction arrays were successfully prepared by the two-step hydrothermal method, and the growth mechanism was systematically studied. We found that the growth process of molybdenum disulfide (MoS_2_) was sensitively dependent on the reaction temperature and time. Through an X-ray diffractometry (XRD) component test, we determined that we prepared a 2H phase MoS_2_ with a direct bandgap semiconductor of 1.2 eV. Then, the photoelectric properties of the samples were studied on the electrochemical workstation. The results show that the ZnO@MoS_2_ heterojunction acts as a photoanode, and the photocurrent reaches 2.566 mA under the conditions of 1000 W/m^2^ sunshine and 0.6 V bias. The i-t curve also illustrates the perfect cycle stability. Under the condition of illumination and external bias, the electrons flow to the conduction band of MoS_2_ and flow out through the external electrode of MoS_2_. The holes migrate from the MoS_2_ to the zinc oxide (ZnO) valence band. It is transferred to the external circuit through the glass with fluorine-doped tin oxide (FTO) together with the holes on the ZnO valence band. The ZnO@MoS_2_ nanocomposite heterostructure provides a reference for the development of ultra-high-speed photoelectric switching devices, photodetector(PD) devices, and photoelectrocatalytic technologies.

## 1. Introduction

The direct bandgap semiconductor ZnO is an n-type semiconductor material with a hexagonal wurtzite structure with a bandgap of 3.37 eV and an exciton beam energy of 60 meV at room temperature. ZnO has excellent dielectric, piezoelectric, photoelectric, and other properties. It has broad application prospects in the fields of photoconductive, piezoelectric, optical waveguide, light-emitting devices, lasers, transparent conductive films, gas sensors, energy conversion, and acousto-optic devices [1,2,3,4,5,6,7,8,9]. Therefore, in recent years, ZnO has received extensive research and attention. ZnO has a wide bandgap, high exciton binding energy, high strength, high hardness, and greater electron mobility than TiO_2_, making it suitable for dye-sensitized solar cells (DSSC). However, the one-dimensional nano-zinc oxide is a single crystal with no grain boundary, and other loss of transport electrons and free electrons are more likely to drift onto the conductive glass. Therefore, the replacement of TiO_2_ with a one-dimensional nanometer ZnO array significantly improves the electron transfer efficiency. The ZnO one-dimensional nanorod arrays are superior to ZnO thin films in terms of optical properties and fast electron transport properties [10,11,12]. When n-type ZnO is combined with a P-type semiconductor, ZnO enhances the visible light absorption as an anti-reflection photon window and also serves as an n-type semiconductor material that generates carriers and provides a depletion layer [13,14] and a built-in electric field. Several synthetic techniques have been used to synthesize ZnO nanomaterials, such as magnetron sputtering, chemical vapor deposition (CVD), hydrothermal synthesis, sol-gel method, pyrolysis process, electrochemical deposition, etc. [15,16,17,18,19,20,21,22,23]. The hydrothermal method operates under mild reaction conditions and does not produce any pollution at all [24,25]. It is an effective method for large-scale preparation of ZnO nanorod arrays.

Transition metal chalcogenides have been extensively studied in catalysts, solid lubricants, batteries, optoelectronic devices, hydrogen production materials, wave absorption, and sensors [26,27,28,29,30,31,32,33,34,35]. As a typical transition metal sulfide, the hexagonal MoS_2_ has a layered structure similar to graphite, and there are strong bonds between atoms in the layer. However, there is only a weak van der Waals force between adjacent layers. Studies have reported that MoS_2_ nanomaterials have many properties not found in bulk materials, such as strong photoluminescence properties (PL) and ferromagnetism [36,37,38,39,40]. At the same time, two-dimensional flake MoS_2_ has excellent physical properties and chemical properties, such as a considerable layered band gap (1.2 to 2.2 eV), large surface area, many edge activation sites, and high in-plane electron mobility [41]. MoS_2_ can achieve tunable electronic/photoelectron performance by controlling film thickness. In addition, MoS_2_ has good chemical stability, and process compatibility is easy to integrate with silicon and use in CMOS logic devices. Ultrathin MoS_2_ nanosheets can effectively suppress short channel effects. Because of these advantages, MoS_2_ nanomaterials have broad application prospects in electronic and optoelectronic devices, photocatalysis, lithium-ion batteries, biology, sensors, spin electronics, and other fields [42,43,44,45,46,47,48]. However, the monolayer’s MoS_2_ absorbs only 5.6% of the bulk material, which greatly limits its use in photodetector (PDs) devices and photocatalysis. In order to meet such applications, nano-molybdenum disulfide is usually combined with other semiconductors with close atomic distance to produce a composite heterostructure. This significantly increases the spectral absorption range and promotes the separation and migration of carriers through the built-in electric fields, thereby, improving the photoelectric response characteristics [49].

In this study, we successfully grow flower-like MoS_2_ on ZnO nanorods following the two-step hydrothermal method forming ZnO@MoS_2_ heterojunction. Among them, ZnO is a wide bandgap (3.37 eV) n-type semiconductor, MoS_2_ nanosheet is a narrow bandgap (1.2 eV) P-type semiconductor [50], and ZnO@MoS_2_ heterojunction contributes to specific charge transfer dynamics and electron-hole pair separation, effectively improving its photoelectric performance [51,52], combined with low-dimensional MoS_2_ film with good on/off current ratio and high carrier mobility. After excitation with 1000 W/m^2^ light, when the bias voltage is 0.3 V, the photogenerated electrons and holes generated in ZnO and MoS_2_ are directly separated. Therefore, the structure exhibit excellent photoelectric response characteristics, as well as stable photoelectric cycle, and, in the future, it is expected to be applied to ultra-high-speed photoelectric light-emitting, PD and photocatalysis, sensors, light-emitting diodes, and other related devices.

## 2. Experimental

### 2.1. Chemicals and Materials

The following chemical reagents were used in the experiment: Deionized water, zinc nitrate hexahydrate(Zn(NO_3_)_2_·6H_2_O), hexamethylenetetramine(HMT), sodium molybdate dihydrate(Na_2_MoO_4_·2H_2_O), thiourea(CH_4_N_2_S), ethanol(C_2_H_5_OH), sodium sulfate anhydrous(Na_2_SO_4_), zinc oxide(ZnO) target material (*φ*60 × 5 mm, purity of 99.99%). All the chemical reagents, in our experiment, were analytically graded and used directly without further purification.

### 2.2. Growing ZnO@MoS_2_ Heterojunction

**Figure 1:**
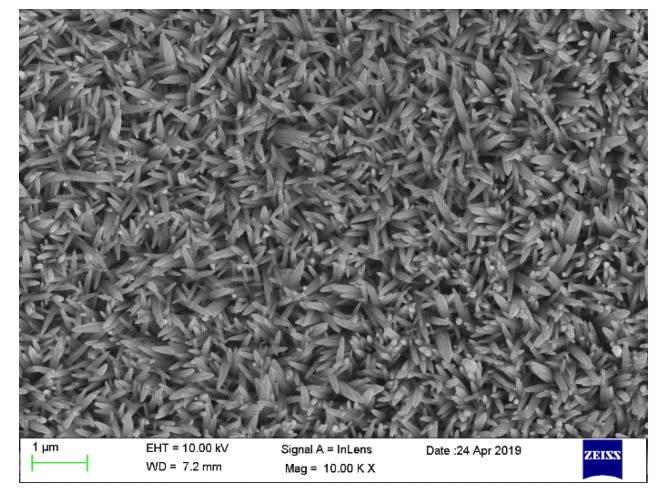
Scanning electron microscopy (SEM) image of ZnO nanorods.

Before growing the ZnO@MoS_2_ heterojunction, ZnO nanorods were first grown by the hydrothermal method. In the experiment, ZnO nanoarrays prepared by hydrothermal and magnetron sputtering were carried out using the preparation process of our previous paper [53]. When the ZnO nanorods were prepared, the concentration of the precursor solution was 40 mmol/L, the reaction temperature was 95 °C, and the reaction time was 4 h. The corresponding SEM image is shown in Figure 1.

The precursors, Na_2_Mo_4_·2H_2_O and CH_4_N_2_S(thiourea), were weighed using a digital balance. The amounts of precursors were 30 and 60 mg, respectively. After weighing, they were placed in a 20 mL aqueous solution for sonication until the solids were all dissolved. We transferred the solution to a Teflon-lined reactor, then, tilted the sample with ZnO nanorods into the reactor (the sample face down), and filled the reactor with an inert gas of Ar. Then, we closed the reaction kettle and put it into the thermostat at set temperatures of 180, 200, and 220 °C. We waited for the temperature to rise to the reaction temperature and started a recording time. The time was 20 h. Then, the above steps were repeated, the reaction temperature was set to 220 °C, and the reaction times were 16 and 24 h, respectively. When the reaction time was reached, the sample was taken out, and we recorded the state of the solution and the surface of the sample after the reaction. Its surface was rinsed with deionized water and placed in a fume hood to dry naturally. Finally, the sample was annealed. We placed the sample in a muffle furnace and heated from 25 °C to 140 °C at a rate of 4 °C/min. We kept it at 140 °C for 80 min, and then, stopped the program. After waiting for the temperature to slowly drop to room temperature (greater than 16 h), we took the sample and set it aside.

### 2.3. Photocurrent Experiment

Electrochemical and photoelectrochemical measurements were performed using a three-electrode system (5.0 × 5.0 × 5.0 cm^3^ quartz-cube beaker), the electrochemical workstation model was the CHI660E. The working electrode used in the test was a sample of FTO@ZnO@MoS_2_ with an effective area of 1 cm^2^, prepared by us. The high potential was connected to MoS_2_. The Na_2_SO_4_ solution of 0.1 mol/L was used as the electrolyte. The counter electrode was a platinum wire with a diameter of 0.5 mm (99.99%, CHI Instruments) and the reference electrode was a saturated calomel electrode (SCE, Shanghai INESA Scientific Instruments, Shanghai, China). The light source was incident from the back of the FTO conductive glass. The light source used in the experiment was a xenon lamp source (CEL-S500/350, Beijing China Education Au-light Co., Ltd., Beijing, China), which was calibrated to 100 mW/cm^2^.

### 2.4. Characterization Techniques

We studied the surface structure and morphology of composite samples by scanning electron microscopy (SEM, JSM-6701F, JEOL Ltd., Tokyo, Japan) and X-ray diffractometry (XRD, D/max-1400, Japanese Science, Japan). The photocurrent and photoresponse of the samples were investigated using an electrochemical workstation.

## 3. Results and Analysis

Since the hydrothermal method in this experiment is carried out in a closed high-temperature, high-pressure reactor, the temperature directly provides power to the reaction process. It also determines the activity of the reaction precursor solution and the pressure of the system, which plays a decisive role in the progress of the reaction and the crystallization rate of MoS_2_. At the same time, temperature determines the solubility of the reactants in solution. Under the same temperature conditions, the higher the saturation achieved by the precursor solution, the faster the growth rate of the crystal. The nucleation rate also directly affect the particle diameter and agglomeration of the sample, thus affecting the morphology, crystallinity, and properties of the prepared MoS_2_. In theory, the higher the reaction temperature, the faster the reaction rate. However, the premise is that the reaction temperature needs to be higher than the critical temperature of the solution so that the liquid phase and the gas phase can coexist. At this time, the reaction proceeds in the direction of generating MoS_2_. Therefore, it is imperative to choose the right temperature for the reaction.

**Figure 2:**
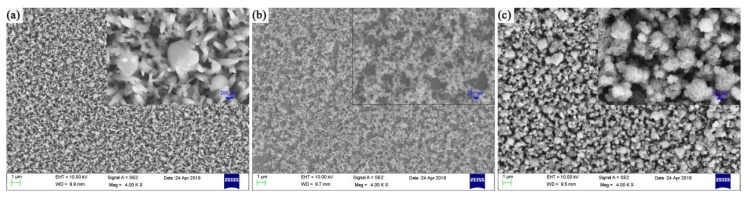
SEM images of the sample at different temperatures under the conditions of reaction time of 20 h and precursor amount of 30 mg. (**a**) Reaction temperature is 180 °C; (**b**) reaction temperature is 200 °C; and (**c**) reaction temperature is 220 °C.

**Figure 3:**
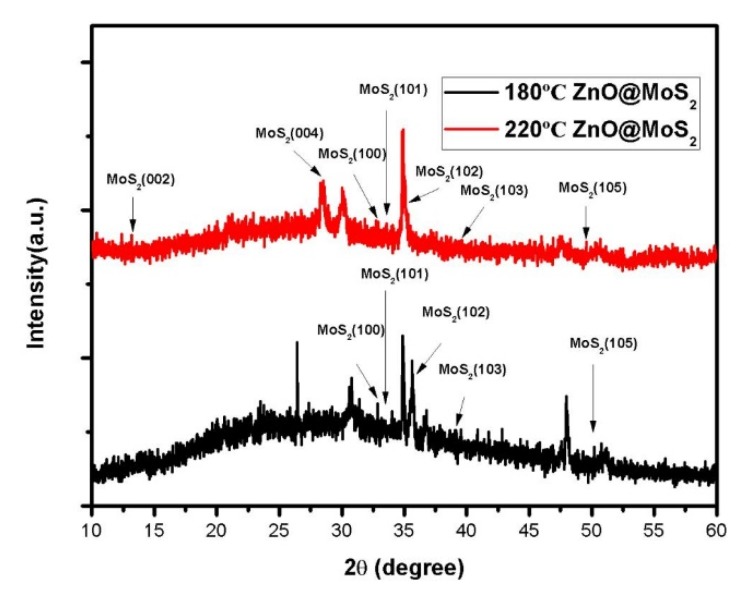
X-ray diffraction patterns of samples at 180 °C and 220 °C.

In order to study the role of the reaction temperature in the hydrothermal growth of MoS_2_ and to determine the optimal reaction temperature, under the condition that the reaction duration is 20 h and the amount of precursor is 30 mg, we set three different temperatures of 180, 200, and 220 °C. Figure 2a is the SEM image when the temperature is 180 °C and Figure 3 is the X-ray diffraction pattern of the samples at different temperatures. In Figure 3, the crystallinity of sample MoS_2_ is very weak at 180 °C, and the diffraction peak appears to be extremely weak, while there is no diffraction peak on the crystal plane (004). As can be seen from Figure 2a, some tiny MoS_2_ particles and a few bulk crystals appeared on the surface of the ZnO nanorods without complete and regular MoS_2_ crystals appearing. The possible reasons are as follows: First, the temperature is too low, and the critical temperature required for the reaction is not reached, therefore, the reaction in the direction of MoS_2_ does not occur substantially; second, the saturation of the reaction solution is low at this temperature and the growth rate is prolonged; third, the liquid phase and the gas phase do not coexist. According to the reaction equation, the gas H_2_S required to be formed in the reaction directly participates in the reaction. Figure 2b is the SEM image when the temperature is 200 °C. When the temperature increases from 180 °C to 200 °C, nucleation sites begin to appear, and form agglomerated spherical MoS_2_. As the temperature increases, the activity of the reactants increases, the size of the resulting sample becomes more extensive, the morphology becomes more regular, the growth distribution becomes more uniform, the reaction rate increases, and the crystallinity of the sample increases. In Figure 3, the (004) crystal plane diffraction peak is significantly enhanced, indicating that the preferred growth direction of the sample is (004) crystal plane [54,55,56]. Figure 2c is the SEM morphological image of the sample at 220 °C. As shown in Figure 2c, when the temperature is raised to 220 °C, the MoS_2_ is almost uniformly grown on the surface of the sample. The MoS_2_ is polymerized into a flower-like material with the largest size and an average diameter of about 800 nm. The exposed crystal has a large specific surface area and a large number of active sites, which is beneficial to the reaction of photoelectric, photocatalytic, and so on.

**Figure 4:**
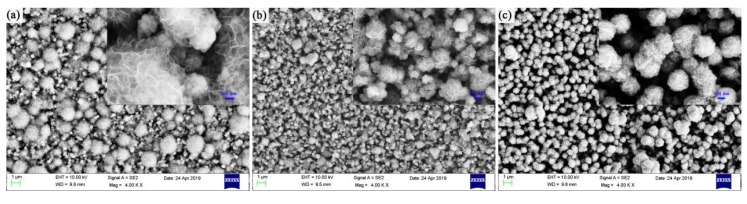
SEM images of the sample at different times at a reaction temperature of 220 °C and precursor amount of 30 mg. (**a**) Reaction time is 16 h; (**b**) reaction time is 20 h; and (**c**) reaction time is 24 h.

In this experiment, we use a neutral solution to grow MoS_2_ in situ on the ZnO substrate. Growth is divided into two steps, one is crystal nucleation, and the other is crystal growth and development. The growing flower-like MoS_2_ can be decomposed into several reaction processes. First, MoS_2_ nucleates and grows into flaky MoS_2_. Then, the flaky MoS_2_ is assembled into a small flower-like shape and continues to grow into a flower-like shape. Finally, it grows into a spherical shape. At the optimum reaction temperature of 220 °C, the precursor solution content is fixed at 30 mg. As time goes by, the crystal shape is more regular. However, excessive time can cause serious grain accumulation. The SEM image of the sample with a reaction time of 16 h is shown in Figure 4a. At this time, since the concentration is sufficient, the reaction has completed the process of nucleation, flaky formation, and assembly into a flower-like shape at a reaction time of 16 hours. Figure 4b is a SEM photograph of the sample having a reaction duration of 20 h. With the extension of time, it is more beneficial to the regularity of crystal morphology. The flower-like shape gradually grows, filling a part of the void, and increasing the density of MoS_2_ in the flower-like shape. However, if the reaction time is extended, the amount of precursor consumed increases, the amount of the remaining precursor decreases, the concentration becomes low, the surface tension between the liquid and the grown crystal is substantial, and the growth rate begins to slow down. Figure 4c is a SEM image of the sample with a reaction time of 24 h. At this time, the sample has grown into a spherical sphere, and the active site is sharply reduced as compared with the flower-like site. Through analysis, if you want to make the flower-like shape last for a long time, you need to increase the concentration. With the rise of concentration, the amount of nucleation sites increases, and the number of flower-like forms is more, which is not quickly filled into a spherical shape. The reason for this could be that the energy required to grow crystals in different orientations is different. Increasing the concentration of the precursor solution reduces the surface tension, increase the molecular potential energy, and select an energy-oriented orientation growth.

**Figure 5:**
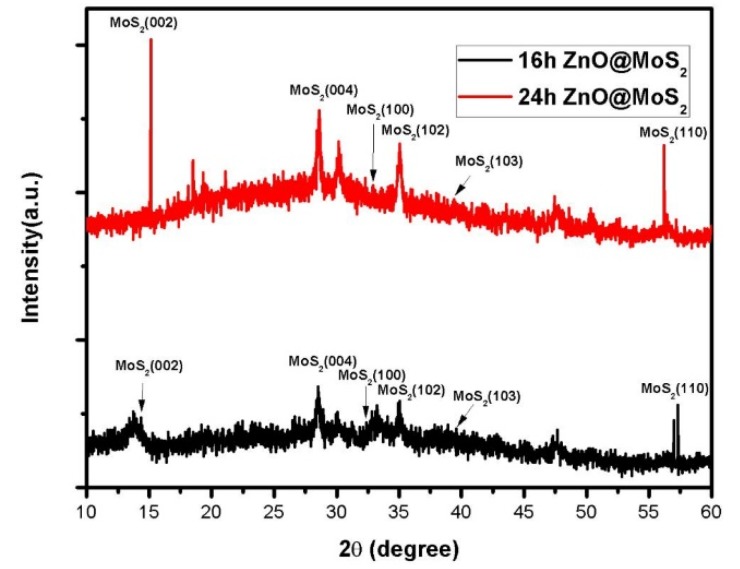
X-ray diffraction pattern of samples at 16 h and 24 h.

Figure 5 shows the X-ray diffraction patterns of samples at 16 h and 24 h. It can be seen from the XRD pattern that, as the reaction time is prolonged, the flaky crystals gradually accumulate in layers and develop toward the bulk material. If the time is too long, severe grain accumulation occurs. Therefore, the (002) crystal plane diffraction peak is enhanced [57,58]. Noticeable diffraction enhancement is found in the direction of (004), (102), and (110), indicating that the crystal grows preferentially in the direction of (004), (102), and (110). At 16 h, the peak intensity of (002), (004), (102), and (110) are significantly weaker than that of the sample at 24 h. And the sample has a very small half-peak at 16 h, indicating that the sample has good crystallinity.

**Figure 6:**
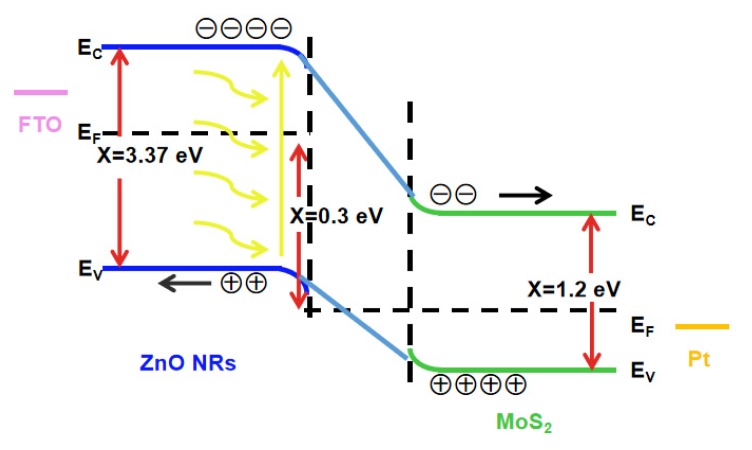
Energy band schematic diagram of ZnO@MoS_2_ receiving light to form photogenerated carriers under an external bias voltage.

The photoelectric test principle is that ZnO nanorods contact with the flower-like MoS_2_ to form a heterostructure, and the Fermi level tends to be consistent [59,60,61]. The heterostructure is used as a photoanode, and the photoelectric response is tested by an electrochemical workstation under a bias voltage of 0.3 V. At this time, a bias voltage is applied in order to ultimately collect electrons and holes produced by the light and separated by the built-in electric field as much as possible. The carriers flow out from the two electrodes and are collected and utilized by an external circuit, as shown in Figure 6.

**Figure 7:**
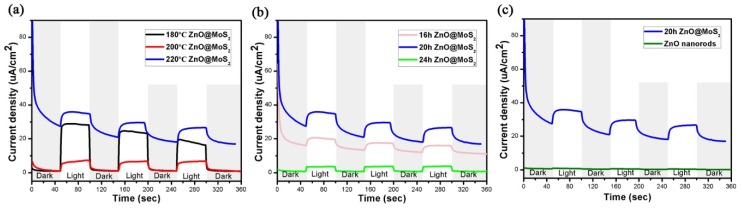
(**a**–**c**) the i-t curve of the sample was prepared in a 0.1 M Na_2_SO_4_ (pH = 6.8) electrolyte under the action of a constant potential of 0.3 V vs. SCE.

Figure 7a shows the transient photoresponse current of ZnO@MoS_2_ nanorods under dark/visible light cycling conditions with a fixed bias voltage of 0.3 V at different reaction temperatures. ZnO@MoS_2_ nanorods are at the temperatures of 180, 200, and 220 °C under the conditions of the same precursor solution content of 30 mg and reaction time of 20 hours. Combined with the above SEM and XRD analysis, when the temperature is increased from 180 to 200 °C, nucleation sites begin to appear, and agglomerated spherical MoS_2_ has formed. With an increase of temperature, the activity of the reactants increases, the size of the sample is large, the morphology becomes regular, the growth distribution is uniform, the reaction rate is increased, and the crystallinity of the sample is improved. The diffraction peak of crystal plane (004) is significantly enhanced, indicating that the sample grows preferentially along the direction of the crystal plane (004). When the temperature is increased to 220 °C, the MoS_2_ grows most evenly on the surface of the sample. The flaky MoS_2_ polymerizes into a flower-like material with the largest size and an average diameter of about 800 nm. The exposed crystal has a large specific surface area and many active sites, which facilitates the reaction of photoelectric, catalytic, and the like. Therefore, at 220 °C, ZnO@MoS_2_ nanorods achieve the highest instantaneous photocurrent under cyclic lighting conditions.

Figure 7b shows transient light response currents of ZnO@MoS_2_ nanorods under dark/visible light cycling conditions with a fixed bias of 0.3 V at different reaction times. The ZnO@MoS_2_ nanorods are at the reaction time of 16 h, 20 h, 24 h under the conditions of the same precursor solution content of 30 mg and reaction temperature of 220 °C. It can be seen from the three repeated cycles that under light conditions, the photogenerated current slowly decreases with time and finally tends to be consistent, which has a degeneracy effect. For each sample, the current under visible light is more significant than that under dark conditions. The reason is that the two-dimensional sheet MoS_2_ and ZnO are direct bandgap semiconductor materials, and the forbidden bandwidth is 1.2 eV and 3.37 eV, respectively. When light illuminates the sample, since ZnO and MoS_2_ receive photons that match the forbidden bandwidth, the electrons on the valence band of the two materials use photon energy to transition to the conduction band, leaving holes in the valence band at the same time. Under external bias, electrons flow into the conduction band of MoS_2_ and out through the external electrode of MoS_2_ [62,63,64,65]. The holes are transferred from MoS_2_ to the ZnO valence band, and together with the holes in the ZnO valence band are transferred to the external circuit through the FTO conductive glass. Therefore, under the condition of no light, the electrons in the valence band of MoS_2_ and ZnO cannot transition to the conduction band because they cannot obtain enough energy. A transient photocurrent cannot be formed because no carriers are generated.

ZnO@MoS_2_ nanorods have the same precursor solution content of 30 mg at the same temperature of 220 °C, and the reaction times are different, which are 16 h, 20 h, and 24 h, respectively. Compared with the other two, the photocurrent of 20 h ZnO@MoS_2_ nanorods is obviously shifted upwards. The reason is that as time goes on, the crystal shape is more favorable, but too long causes severe grain accumulation. When the reaction lasted for 16 hours, the ZnO@MoS_2_ nanorods completed the process of nucleation, sheet formation, and assembly into a flower-like shape. However, due to the short time, the sample does not reach the best shape, and the specific surface area has room for growth. As time goes by, the crystal shape is more regular. The flower-like shape gradually grows, filling part of the void, and increasing the density of MoS_2_ in the flower-like form. At this time, it is 20 h ZnO@MoS_2_ nanorods. However, if the reaction time is prolonged, the concentration of precursor becomes lower and lower. As the surface tension between the liquid and the crystals increases, the growth rate begins to slow down, and the final sample grows to a spherical shape. The active site is drastically reduced, which reduces the effective contact area between the carrier and the electrolyte, and the resistance of the loop is enormous. At the same time, as compared with the flower-like materials, the optical path length, optical absorption rate, and available photons are reduced. Therefore, the photocurrent is lowered. In summary, ZnO@MoS_2_ nanorods at 220 °C for 20 h achieve the highest instantaneous photocurrent. Figure 7c is the transient photoresponse current of the ZnO@MoS_2_ sample and ZnO nanorods without growing MoS_2_ under a fixed biased dark/visible light cycling condition of 0.3 V. It can be seen from Figure 7c that the photoelectric cycle current of ZnO nanorods grown with MoS_2_ is much higher than that of ZnO nanorods without MoS_2_ because MoS_2_ and ZnO form a space charge region. Under light, there are many movable electron-hole pairs in the space charge region, which increase the photocurrent.

**Figure 8:**
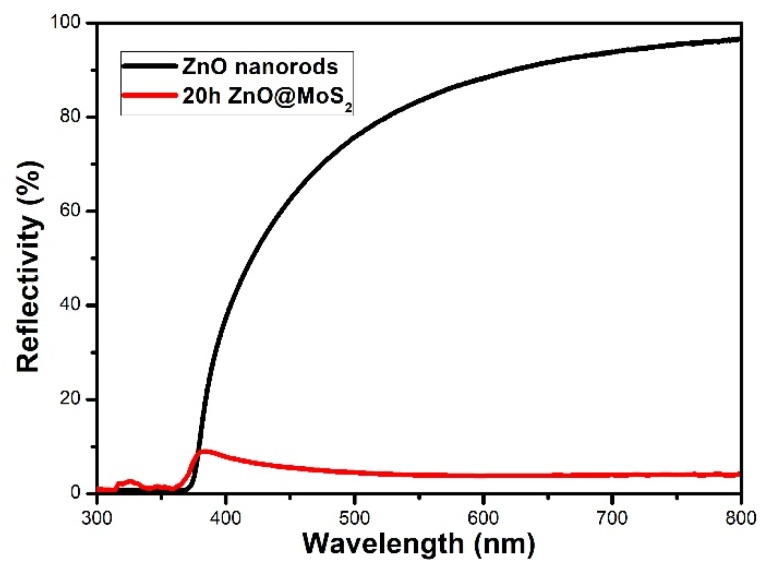
Reflection spectrum of 20 h ZnO@MoS_2_ and ZnO nanorods.

Figure 8 compares the reflectivity of the ZnO@MoS_2_ sample with a reaction temperature of 220 °C and a reaction time of 20 h, with that of ZnO nanorods without MoS_2_ growth. The experimental results show that the reflectivity decreases with the growth of MoS_2_ in the visible region but has no significant change in the ultraviolet region. As a wide band gap material, ZnO itself has a very high refractive index (2.019). This means that the absorption of the ZnO@MoS_2_ sample in the visible region increases significantly. This echoes the above explanation of photocurrent. When the ZnO@MoS_2_ sample is under simulated solar illumination, ZnO and MoS_2_ receive photons that match the forbidden band, and the electrons on the valence band use the photon energy to jump to the conduction band.

**Figure 9:**
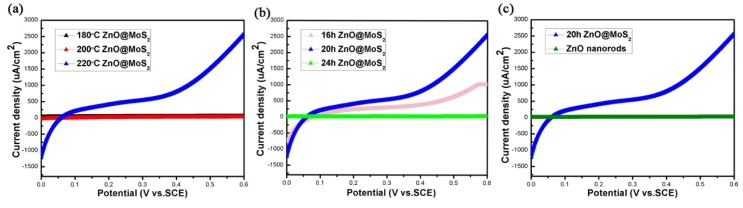
(**a**–**c**) LSV scanning of the prepared sample in a 0.1 M Na_2_SO_4_ (pH = 6.8) electrolyte under a simulated sunlight of 100 mW/cm^2^ with a scanning speed of 15 mV/s.

Correspondingly, we placed the ZnO@MoS_2_ nanorods under 100 mW/cm^2^ simulated sunlight and performed linear sweep voltammetry (LSV) scanning in 0.1 M Na_2_SO_4_ (pH = 6.8) electrolyte to draw V-I curve with the scanning rate of 15 mV/s. The test results are shown in Figure 9a–c. Under the bias condition of 0 to 0.6 V, no breakdown phenomenon occurs. At the voltage of 0.6 V, the ZnO@MoS_2_ nanorods at 220 °C for 20 h reach the extreme value of 2.566 mA. Moreover, the photocurrent of the sample continues to rise. As the bias voltage increases, the current value continues to rise, and no breakdown occurs. Moreover, three repeated cycles indicate that the photocurrent under the light condition has excellent stability, good repeatability, and no degradation. The photocurrent value of the device is increased to saturation within 10 s. Therefore, the structure exhibits excellent photoelectric response characteristics as well as a stable photoelectric cycle.

## 4. Conclusions

In summary, the growth of flower-like MoS_2_ on ZnO nanorods is accomplished by the hydrothermal method. The effects of temperature and time on the preparation of MoS_2_ were investigated by SEM and XRD. The preparation process parameters with the best morphology and the best performance are defined to be a reaction temperature of 220 °C, a precursor amount of 30 mg, and a reaction time of 20 hours. Under the action of constant potential 0.3 V vs. SCE, in the 0.1 M Na_2_SO_4_ (pH = 6.8) electrolyte, the ZnO@MoS_2_ nanorods at 220 °C for 20 h obtain the maximum transient photoresponse current under dark/visible light cycling conditions with excellent photocurrent stability (photoresponse stability) in repeated cycles, excellent repeatability, and no degradation. Under the simulated sunlight irradiation of 100 mW/cm^2^, and the bias voltage condition of 0 to 0.6 V, the current value of ZnO@MoS_2_ nanorods at 220 °C for 20 h continues to rise with an increase of the bias voltage, does not exhibit breakdown, and reaches 2.566 mA photocurrent. This study shows that the structure exhibits good photoelectric response characteristics, as well as stationary photoelectric cycling. In the future, it is expected to be applied to ultra-high-speed photoelectric light-emitting, PD and photocatalysis, sensors, light-emitting diodes, and other related devices.
